# Successful Multimodal Therapy with Intracerebral Liposomal Amphotericin B and Systemic High-Dose Isavuconazole in Proven Disseminated Aspergillosis

**DOI:** 10.3390/jof9030327

**Published:** 2023-03-07

**Authors:** Simon Feys, Franceska Dedeurwaerdere, Katrien Lagrou, Jeroen Van Lerbeirghe, Dries Deeren

**Affiliations:** 1 Medical Intensive Care Unit, UZ Leuven, 3000 Leuven, Belgium; 2 Department of Microbiology, Immunology and Transplantation, KU Leuven, 3000 Leuven, Belgium; 3 Department of Pathology, AZ Delta, 8800 Roeselare, Belgium; 4 Department of Laboratory Medicine and National Reference Center of Mycoses, UZ Leuven, 3000 Leuven, Belgium; 5 Department of Neurosurgery, AZ Delta, 8800 Roeselare, Belgium; 6 Department of Hematology, AZ Delta, 8800 Roeselare, Belgium

**Keywords:** aspergillosis, cerebral aspergillosis, *Aspergillus* empyema, amphotericin B, isavuconazole, *Aspergillus flavus*

## Abstract

We report the case of a 32-year-old man receiving chemotherapeutics for an acute B-lymphoblastic leukemia who developed proven cerebral and pulmonary aspergillosis with *Aspergillus flavus*. Because of progressive fungal disease with neurological deterioration despite adequate systemic antifungal therapy and surgical debridement, intracerebral administration of liposomal amphotericin B was initiated at 5 mg twice weekly. This led to improvement of the cerebral infection. Surgical debridement of a pleural *Aspergillus* empyema was necessary, and pleural trough level of isavuconazole was found to be subtherapeutic despite adequate blood trough levels, which led us to increase the dose of isavuconazole. We conclude that intralesional amphotericin B might be beneficial at 5 mg twice weekly in cerebral aspergillosis if systemic antifungals and surgical debridement fail. In *Aspergillus* empyema, measurement of pleural isavuconazole trough levels should be considered.

## 1. Introduction

Invasive aspergillosis is one of the most frequent invasive fungal infections, with an estimated annual incidence of approximately 250,000 cases each year [1]. This was recently acknowledged by the World Health Organization by naming the most common cause of invasive aspergillosis (*Aspergillus fumigatus*) one of the top four priority fungal pathogens to guide research, development, and public health action [2]. Invasive aspergillosis typically affects severely immunocompromised patients as those with risk factors defined by the European Organisation for Research and Treatment of Cancer and Mycosis Study Group Education and Research Consortium [3]. While *Aspergillus* typically finds its primary point of entry in the lungs, and thus most often causes invasive disease in this organ, hematogenous spread is possible and may affect brain, kidneys, and other organs. Especially if the invasive disease arises in the brain, prognosis is poor, as the antifungal treatment may have difficulty reaching the fungus due to the blood-brain barrier and the damage caused by invasive hyphae may cause irreversible neurological deficit [4,5]. In cases in which a pulmonary focus is (initially) not apparent, isolated cerebral infection may pose a diagnostic challenge and often requires biopsy to obtain a diagnosis of invasive aspergillosis.

Azoles typically are the first option to treat invasive aspergillosis but are notorious for their side effects and drug-drug interactions. Recently, isavuconazole was shown to be comparable to the current first-line antifungal voriconazole, but had a more favorable drug-related toxicity profile [6]. Both voriconazole and isavuconazole are considered first-line antifungals to treat invasive pulmonary aspergillosis in patients with hematological malignancies [7]. Amphotericin B used to be the mainstay of therapy but is now only used as second line of therapy since the advent of voriconazole. Cerebral disease may warrant surgical debridement to achieve disease control, which inherently causes often debilitating damage to brain tissue. Other salvage strategies typically resort to immunomodulation (for instance cytokines such as recombinant interferon-gamma or immune checkpoint inhibitors like anti-PD1), although experience with these agents is very limited [8,9]. 

In this report, we describe the case of a neutropenic patient receiving intensification chemotherapy for acute lymphoblastic leukemia who developed cerebral and pulmonary aspergillosis requiring multimodal therapy with systemic isavuconazole, surgical debridement, and intralesional amphotericin B. We resorted to an intracerebral amphotericin B administration regimen suggested by Schauwvlieghe et al., that was never reported in practice before [5]. Moreover, we report the first measurements of pleural isavuconazole levels in *Aspergillus* empyema. 

## 2. Detailed Case Description

A 32-year-old man arrived at the emergency department (ED) with a one-day history of progressive weakness in the right leg. He had been suffering from acute B-lymphoblastic leukemia (B-ALL) for eight months, for which he had recently received a 28-day course of intravenous blinatumomab after intensive remission-induction therapy with complete remission of the disease. An intensification scheme with dexamethasone, vinblastine, doxorubicin, PEG-asparaginase, and methotrexate (the latter also intrathecal) was started three weeks prior to the arrival at the ED. The patient developed neutropenia (nadir as outpatient 0.03 × 10^9^/L, normal absolute neutrophil count range 1.7–6.8 × 10^9^/L), documented for the first time five days before hospitalization. Lipegfilgastrim and a prophylactic course of ciprofloxacin was commenced. Prophylaxis for invasive mold disease was not initiated since the duration of neutropenia was predicted to be less than seven days. Two days prior to hospitalization, the patient was seen on day clinic with persistent neutropenia (30/µL) and oral mucositis for which treatment with fluconazole was initiated.

**Figure 1:**
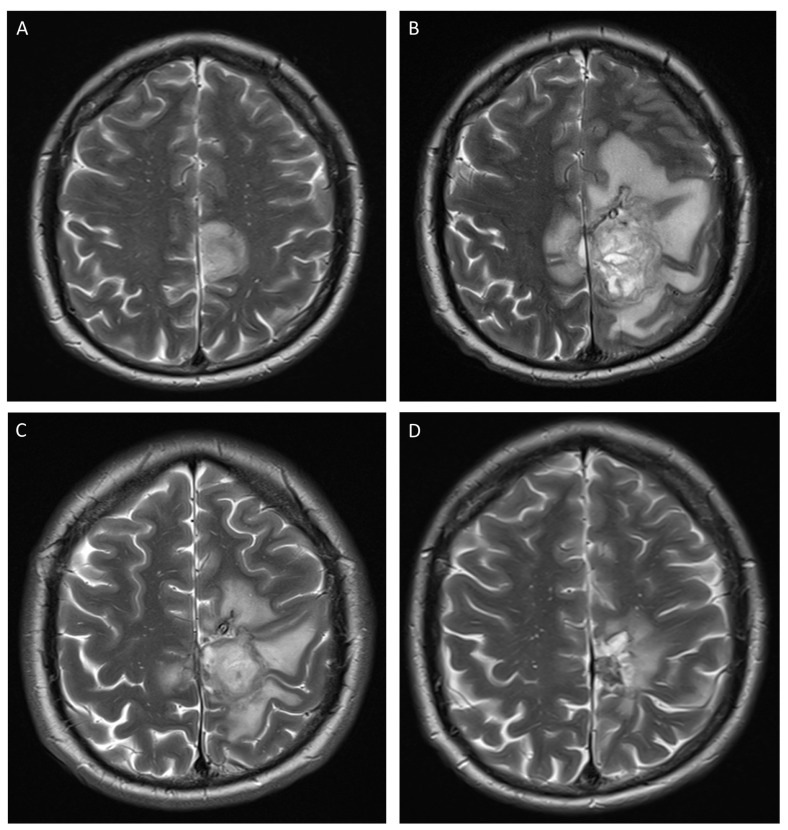
Serial T2-weighted MRI images. (**A**): Day 1 of admission. Hyperintense lesion with maximal diameter of 35 mm in the left frontal lobe with limited extension to the right frontal lobe. (**B**): Day 22 of admission. Increased lesion with a maximal diameter of 55 mm and important perilesional edema with small midline shift. (**C**): Day 70 since hospitalization, six weeks after start of intralesional liposomal amphotericin B. Decreased lesion with a maximal diameter of 43 mm and reduced perilesional edema without midline shift. Ommaya drain visible within the lesion. (**D**): Nine months since hospitalization. Small residual lesion with maximal diameter of 14 mm, with hemosiderin depositions and surrounding gliosis.

Upon arrival at the ED, a complete paralysis with retained sensory function was noted in the entire right lower limb. The patient reported no other complaints and clinical examination was otherwise normal. Blood tests showed a persistent neutropenia (0.03 × 10^9^/L), along with a CRP of 141 mg/L (normal range <5.0 mg/L). Brain computed tomography (CT) followed by magnetic resonance imaging (MRI) (Figure 1A) was performed and showed a nodular infiltrative lesion medially in the left frontal lobe, with limited infiltration of the right frontal lobe. A lumbar puncture showed a slightly raised cerebrospinal fluid (CSF) protein level (92 mg/dL), but normal cytosis and glucose level. Polymerase chain reaction (PCR) for multiple pathogens (including Toxoplasma), along with culture, was performed on the CSF, but the outcome was negative. Galactomannan was not assessed on CSF. 

**Figure 2:**
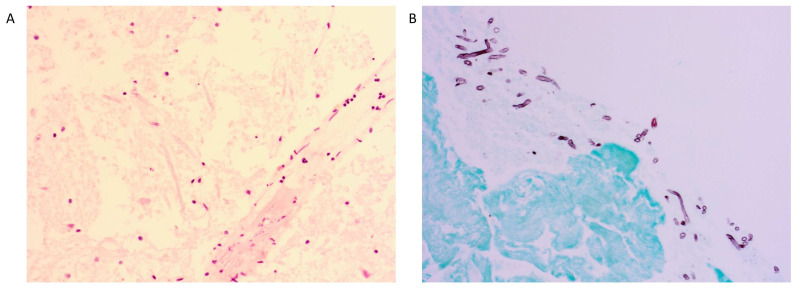
(**A**): Hematoxylin and eosin staining of the cerebral abscess biopsy specimen showing necrotizing glial tissue with invading hyphae. (**B**): Grocott staining of the pleural biopsy specimen showing chronic organizing pleuritis with invading hyphae.

The patient was admitted to the hematology ward and therapy with liposomal amphotericin B was commenced for the greatest possible fungal coverage, along with cefepime, clindamycin, pyrimethamine, and levofolic acid; the last three treatments were interrupted once Toxoplasma PCR and serology came back negative. As the patient started to show rapidly progressing paresis of the right upper limb during the first day of admission, a short trial of dexamethasone was started to tackle possible brain edema, but was discontinued after two days (cumulative dose 32 mg) since there was no effect on disease progression. On the third day since admission, the neutropenia had resolved. A neurosurgical stereotactic biopsy of the lesion was performed. Direct microscopic examination on abscess fluid revealed hyphae, but culture of the fungus from the biopsy material (and blood culture) was not successful and PCR on abscess fluid for both *Aspergillus* and mucorales was negative (the latter also on serum). Galactomannan was not tested on the abscess fluid. Histology showed necrotizing glial tissue with multiple invasive hyphae (Figure 2A). In the meantime, serum galactomannan turned out positive (optical index 0.839), and isavuconazole was added to the treatment with amphotericin B to assure better coverage for invasive aspergillosis. Isavuconazole was chosen over voriconazole given its better drug-related toxicity profile. A chest CT scan revealed bilateral multiple pulmonary nodular lesions, possibly due to invasive fungal disease, despite lack of respiratory symptoms.

Since the paresis of the right upper limb further progressed, surgical debridement of the abscess was performed on the sixth day since hospital admission. Culture on the debrided material showed growth of *Aspergillus flavus* (confirmed by b-tubulin sequencing), leading to the diagnosis of proven disseminated aspergillosis. Antifungal susceptibility testing showed a minimum inhibitory concentration (MIC) of 2.0 mg/L to amphotericin B and sensitivity to isavuconazole (MIC 1.0 mg/L) (Table 1).

Despite the debridement and adequate systemic antifungal therapy, the patient developed a fever on day fifteen of hospitalization, along with progressive neurological deficit and oxygen need. New CT imaging showed important increase in size of the abscess and a complete consolidation of the left and right lower lobes with parapneumonic effusion on the left side, along with general growth of the previously described nodular lesions. 

To address the cerebral abscess, a new surgical debridement was performed on the fourteenth day of hospitalization and an Ommaya reservoir placed subcutaneously with its drain located in the abscess, with the aid of neuronavigation. The lung and pleural involvement were addressed with antibiotic coverage (piperacillin-tazobactam, which was switched to meropenem after two days) to target possible bacterial superinfection and serial thoracocenteses, which showed exudative fluid without culturable organisms. 

Despite these interventions, the neurological and respiratory condition of the patient worsened with ongoing fever, progressive right facial nerve paresis, bradyphrenia, and development of pleural pain. New cerebral MRI (Figure 1B) and chest CT showed that the disease was progressing with further growth of the cerebral abscess (with development of a midline shift) and the pulmonary nodules and extensive left-sided pleural fluid. 

Administration of intralesional 5 mg amphotericin B via the Ommaya reservoir was initiated on day 23 of hospitalization and was performed twice weekly. Systemic amphotericin B was discontinued. A video-assisted thoracoscopy was performed on day 27 and showed empyematous effusions, which were drained and talcaged. Pleural biopsy displayed the same fungal findings as seen in the cerebral biopsy, while the culture remained negative (Figure 2B). Isavuconazole trough level was measured from the pleural fluid obtained during the thoracoscopy and was 1.1 mg/L, despite adequate serum levels >3 mg/L at the time of the thoracoscopy; the dose of isavuconazole was increased from 200 mg to 300 mg daily when this result became available.

The patient’s neurological status started to improve once intralesional amphotericin B administrations had commenced. Oxygen supplementation was halted and fever subsided five and seven days, respectively, after the thoracoscopy. The patient was discharged from the hematology ward to a rehabilitation unit 56 days after his arrival at the ED. Intralesional amphotericin B was administered twice weekly for a total of six weeks, after which the Ommaya reservoir was removed because of a bacterial superinfection with *Staphylococcus epidermidis* and possibly *Acinetobacter baumannii*; the latter, however, only became positive in blood culture. The patient received intravenous vancomycin and meropenem for fourteen days to treat the bacterial superinfection through a short readmission at the hematology ward. Eventually, he could leave the rehabilitation unit 126 days after initial presentation. By that time the complete right-sided hemiplegia had resolved to a mild right-sided loss of strength along with a persistent paralysis of the right foot. 

The patient received monthly brain imaging (CT or MRI, Figure 1C,D) which showed decrease of the volume of the lesion until seven months after the initial symptoms, after which only the old cyst wall persisted. Twelve months after the initial symptoms, the patient had a successful allogeneic hematopoietic stem cell transplant. At the time of writing this report, 30 months after the initial symptoms, the patient is still receiving isavuconazole daily. There have been no new arguments for invasive fungal disease. 

## 3. Discussion

We describe a complex case of disseminated aspergillosis in an immunocompromised patient. This is the first report describing cerebral intralesional amphotericin B administration at a dose of 5 mg twice weekly, and the first to describe isavuconazole trough level measurement from pleural fluid. 

This case was therapeutically challenging giving the failure of systemic antifungal therapy combined with aggressive surgical debridement to contain the cerebral abscess, leading to an extremely dismal prognosis. While systemic antifungals such as amphotericin B or isavuconazole may penetrate the central nervous system and reach high concentrations in normal cerebral tissue, they are known to have trouble reaching sufficient concentrations in established cerebral fungal abscesses [10,11,12,13]. Moreover, more aggressive surgical debridement would at least have caused a complete and persistent right-sided hemiplegia [14,15]. This prompted us to start intralesional liposomal amphotericin B administration via an Ommaya reservoir, in conjunction with continued systemic therapy with isavuconazole. A small number of cases have reported intralesional amphotericin B administration for cerebral aspergillosis, administering liposomal amphotericin B at a dose of 1 to 3 mg once weekly [5,16,17]. We chose to administer the antifungal twice weekly and at a dose of 5 mg (a scheme that has been proposed by Schauwvlieghe et al. but had never been tested in practice) [5]. We considered that the wash-out effect of the antifungal concentration by the daily production of 500 mL CSF would only be mild since the abscess had no clear connection to the CSF, thus not requiring daily administration of the antifungal. Moreover, this strategy reduced the risk of contamination and superinfection of the Ommaya reservoir. We preferred to dose higher than the previously described 1 or 3 mg per administration for cerebral aspergillosis, given the intermediate susceptibility of the pathogen to amphotericin B in this case. Toxicity due to intralesional amphotericin B was not noted. Limitations of our case include the lack of monitoring of amphotericin B levels on the abscess fluid and the fact that we cannot be completely sure of the added value of intralesional liposomal amphotericin B in this case as the patient received systemic isavuconazole as well. However, since the abscess was progressing during three weeks of adequate systemic therapy and two surgical debridements and the situation only stabilized and improved after the start of the intralesional administration, we consider this as a signal of effectiveness of this treatment modality. Caution should be used when resorting to intralesional administration of drugs through implanted reservoirs, as they are prone to superinfection that may lead to meningitis.

We report the first measurement of an isavuconazole trough level from pleural fluid. This showed a three-times lower level (at a daily dose of 200 mg for four days) than the trough level measured in serum three days earlier (the latter after seven days of uninterrupted treatment at a dose of 300 mg, with similar trough levels as those obtained in the SECURE trial) [6]. The penetration level of isavuconazole in pleural fluid thus seems to be lower than what is seen in voriconazole [16,18]. The clinical impact of the assessed drug levels in the pleural fluid is unclear, as there are currently no formal specific guidelines that help governing therapeutic drug monitoring with isavuconazole (given the lack of a known therapeutic range that should be aimed for). The dose increase of isavuconazole could have impacted the cerebral infection as well, although we deem the effect of initiation of intralesional amphotericin B as the main reason for neurological improvement, given that this amelioration had already started prior to the dose increase of isavuconazole. We did not perform a serum trough level measurement of isavuconazole on the day of the pleural trough level measurement, nor did we perform serial pleural trough levels after increase of the dose of isavuconazole (since no more pleural taps were performed after the thoracoscopy of the patient). Further research investigating the penetration of isavuconazole in the pleural cavity and in empyema is warranted, and the therapeutic efficacy of drug monitoring in non-serum body fluids needs definitive investigation. 

## 4. Conclusions

This case supports use of intralesional amphotericin B at 5 mg twice weekly in cerebral aspergillosis in which systemic antifungals and surgical debridement fail. Measurement of pleural isavuconazole trough levels should be considered in Aspergillus empyema. 

## Figures and Tables

**Table 1:** Minimum inhibitory concentrations (MIC) of the isolated *Aspergillus* strain. EUCAST guidelines were used to guide the determination of susceptibility to the antifungals.

Antifungal	MIC
Amphotericin B	2.0 mg/L
Voriconazole	1.0 mg/L
Posaconazole	0.25 mg/L
Itraconazole	0.25 mg/L
Isavuconazole	1.0 mg/L

## Data Availability

The data presented in this case report are available within the article.
